# Osteogenic and Biomedical Prospects of Hafnium and Its Compounds: A Scoping Review

**DOI:** 10.7759/cureus.54054

**Published:** 2024-02-12

**Authors:** Vaishnavi Rajaraman, Padma Ariga, Deepak Pandiar, Saravanan Sekaran, Karthikeyan Ramalingam

**Affiliations:** 1 Prosthodontics, Saveetha Dental College and Hospitals, Saveetha Institute of Medical and Technical Sciences, Saveetha University, Chennai, IND; 2 Oral Pathology and Microbiology, Saveetha Dental College and Hospitals, Saveetha Institute of Medical and Technical Sciences, Saveetha University, Chennai, IND

**Keywords:** transition metals, radiotherapy (rt), implant osseointegration, hafnium compounds, hafnium, biomedical

## Abstract

The direct engagement of hafnium (Hf) in biological processes or its critical function in living things is not well understood as of now. Unlike key elements like oxygen, carbon, hydrogen, and nitrogen, which are necessary for life, Hf is not known to have any biological activities or functions. It is essential to acknowledge that scientific research is ongoing and that new findings may have been made. This systematic review aimed to aggregate and analyze the studies that discuss biomedical applications of Hf metal. This systematic review was conducted following the guidelines of the Preferred Reporting Items for Systematic Reviews and Meta-Analyses (PRISMA) Statement. The following search strategy was used: two independent researchers conducted electronic searches in databases including PubMed, Embase, Cochrane Database of Systematic Reviews, and Google Scholar. The search was conducted up to August 2023 using the Medical Subject Headings (MeSH) terms “transition elements,” “hafnium,” and “biomedical research.” Boolean operators “AND” and “OR” were used to refine the search. Electronic databases, along with hand searches, identified a total of 38 studies. The various database searches resulted in a total of 38 studies, of which 12 were excluded as duplicates, and five were unavailable for full-text data. The remaining 21 full-text articles were then assessed for their eligibility based on the inclusion and exclusion criteria, and finally, a total of 12 studies were included in the present systematic review. Among the 12 chosen studies, six were on cancer-related targeted radiotherapy or chemoradiotherapy, five were on bone or apatite-forming capabilities, and one was on the treatment of inflammatory bowel disease. The common outcome measures included cell proliferation, osteoblast formation, radiotherapy intensification, and immunotherapy. This review outlines an overall picture of the biomedical uses of Hf metal, a transition element, as a potent biomaterial. In conclusion, this transition element, Hf, has some promising scope in the fields of biomedicine, with a special focus in terms of cancer radiotherapy and osteogenic capabilities.

## Introduction and background

Transition elements are important facets of dentistry. These classes of metals are valuable in dental products and procedures due to their distinctive features [1,2]. A few metals of this group are titanium, zirconium, cobalt-chromium, nickel-titanium, gold, copper, and silver. These transition elements offer a range of properties that cater to specific dental applications such as restorations, orthodontics, and implants [3,4]. The selection of a particular material depends on factors like the patient's needs, aesthetic preferences, and the functional requirements of the dental restoration or treatment. As dental materials and technology continue to advance, new applications of transition elements may emerge in dentistry.

The transition metal hafnium is renowned for its resistance to corrosion and high melting point. It is employed in nuclear reactors, aerospace alloys, and electronic applications because of its resistance to extreme temperatures and ability to maintain stability in a variety of settings. It is frequently found in zirconium minerals. As noted in the previous literature, lanthanide-series compounds incorporating hafnium have been investigated for potential biomedical applications [5-7]. Typically, the focus of these investigations is on the biocompatibility and security of materials containing hafnium for use in implants, medical devices, and other healthcare applications [8]. As a potential component to improve osseointegration in dental implantology, hafnium shows huge promise [9-11]. It is a desirable choice for covering dental implants because of its special qualities, which include biocompatibility, resistance to corrosion, and the capacity to produce bioactive oxide layers [12].

Even though research is still in progress, hafnium's contribution to osseointegration may have a major positive impact on the efficacy and durability of dental implant procedures. To further investigate the possible uses of hafnium in dentistry and convert research findings into clinical practice, it is essential that material scientists, dental practitioners, and researchers work together. In this context, hafnium has been used in various studies before to check its osseointegration potential and tissue compatibility with other lanthanide metals and showed promising results. This review aims to scrutinize the studies that evaluated biomedical applications of hafnium metal.

## Review

Material and methodology

This review was reported in accordance with the Preferred Reporting Items for Systematic Reviews and Meta-Analyses (PRISMA) Statement guidelines [13]. The primary objective of this review was to evaluate the biomedical applications of hafnium. The period of the included studies was extended. An electronic database search identified a total of 38 studies. A strategy was planned for this scoping review, and the research question was formulated (Table 1).

**Table 1:** Search strategy for the research question in this scoping review

	Search strategy
Domain	Description
Population	Transition metals
Intervention	Hafnium
Outcome	Biomedical applications

Search Strategy

Two researchers independently conducted electronic searches in databases including Embase, Cochrane Database of Systematic Reviews, PubMed, Scopus, Web of Science, and Google Scholar. The search was conducted up to August 2023 with the terms “transition elements,” “hafnium,” and “biomedical research.” “AND” and “OR” Boolean operators were used to refine the search. The search strategy in PubMed yielded 19 articles.

**Figure 1:**
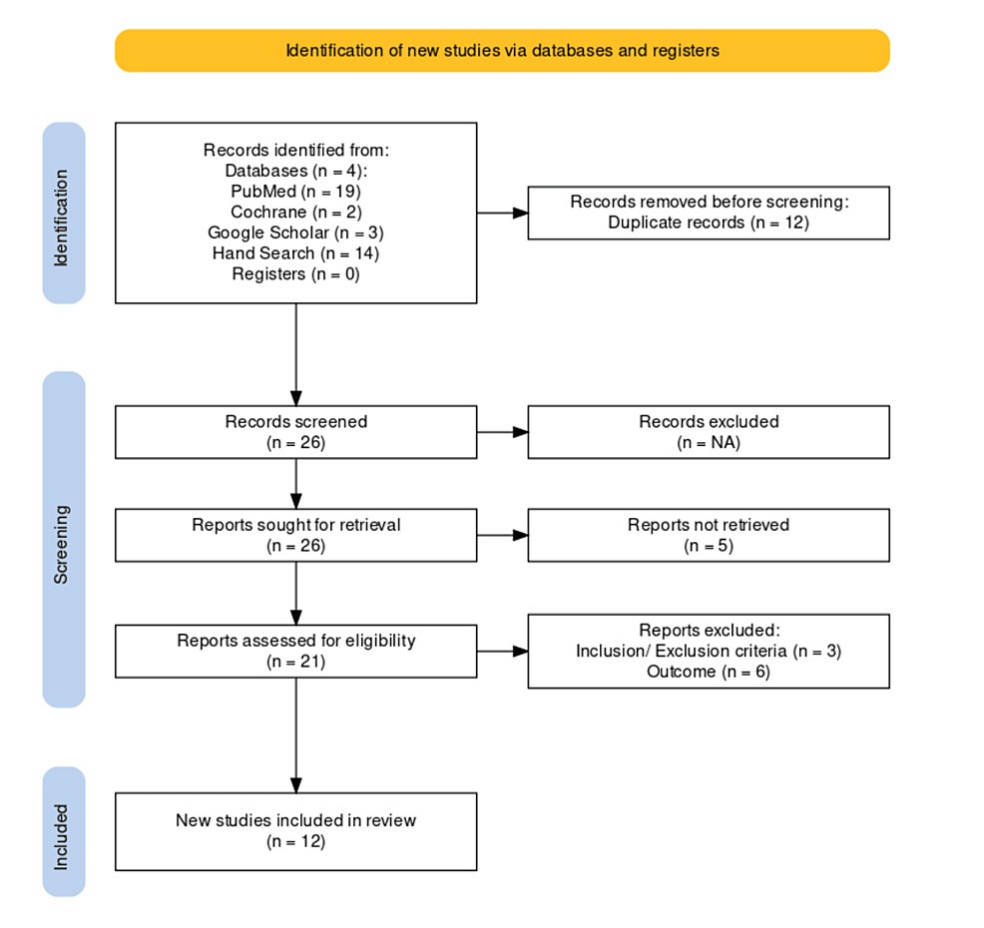
Preferred Reporting Items for Systematic Reviews and Meta-Analyses (PRISMA) flow chart describing the inclusion of studies for this systematic review

An advanced search of the Cochrane search engine was done, and the search yielded two clinical trials. Three articles were retrieved from the Google Scholar engine, and a hand search yielded 14 results. An initial search was performed with the abovementioned keywords and databases. Duplicates were excluded, and studies were screened further. Titles and abstracts of the non-duplicate citations were independently screened in a standardized manner by two calibrated reviewers (VR and PA) for potential inclusion in this review. The remaining included articles were then obtained in full text and then screened, excluding studies as per the inclusion and exclusion criteria, independently by both reviewers. Cohen’s kappa statistic was used to evaluate the agreement between the two reviewers. Any disagreement between the two reviewers was resolved by discussion. Finally, 12 articles were included for data extraction (Figure 1).

Inclusion Criteria

This review included studies meeting specific criteria: those that provided results in terms of biomedical applications or biomedical research, published in English, focused on animal studies, and relevant to hafnium and related compounds.

Exclusion Criteria

Excluded were case reports, case series, and reviews or literature articles, studies that do not report biomedical applications, are irrelevant to hafnium, or do not have full text available.

Focused Question

The focused question was, “Does hafnium have biomedical applications or not?”

Statistical Analysis

A meta-analysis could not be performed for this review as the study results were heterogeneous and non-parametric.

Results

The searches in various databases resulted in 38 studies in total, of which 12 were excluded as duplicates, and five were unavailable for full-text data. The remaining 21 articles were then assessed based on exclusion and inclusion criteria for their eligibility (Table 2), and 12 studies were included in the present review [14-22].

**Table 2:** Studies excluded in this review and the reason for their exclusion

Author, year	Reason for exclusion
Il Song Y et al., 2011 [14]	Outcome: Water and ion penetration in flexible bioelectronic systems
Villa I et al., 2018 [15]	Outcome: Cellular imaging
Reszka P et al., 2019 [16]	Outcome: The materials were assessed for surface morphology.
Verry C et al., 2019 [17]	Review article
Khalladi N et al., 2019 [18]	Exclusion criteria: French article
McGinnity et al., 2021 [19]	Outcome: Synthesis of intervention, not applications
Sebti et al., 2022 [20]	Outcome: Morphological, optical, and photoluminescence properties
Ren HM et al., 2022 [21]	Outcome: Mechanical properties measured
Liu N et al., 2023 [22]	Inclusion criteria: Intervention modified

Among the 12 chosen studies, six were on cancer-related targeted radiotherapy (RT) or chemoradiotherapy, five were on the bone or apatite forming capabilities, and one was on the treatment of inflammatory bowel disease. Most of the studies were based in China; one was a multicenter study, and the other studies were from Japan, Poland, Russia, and India. The common outcome measures included cell proliferation, osteoblast formation, RT intensification, and immunotherapy.

Geographic Distribution

In the current research, the geographic distribution of the study centers is widespread. Of the included studies, five are from China, two studies from Russia and India each, one study from Japan and Poland each, and one from a multicenter study [23,24]. Due to the distribution of studies in a wide geographic range, the results can be extrapolated with minimal risk of bias.

Characterization of Intervention

The articles included in the research studied various forms of hafnium and its compounds. Of these, five studies had hafnium oxide as its intervention, one had hafnium and its alloyed form, one had a coating of hafnium, and the rest had multiple alloyed or compounded forms [15,25]. As this research included all forms of hafnium and its compounds in its intervention, there is no bias regarding the same. Previous studies have had a similar compilation of hafnium and related compounds [26,27].

Outcomes Measured

Among the 12 studies included, six were on targeted RT [28-33] or chemoradiotherapy for cancer, five on the apatite forming abilities, and one on efficacy in the treatment of inflammatory bowel disease. The current research aims to find all the biomedical applications of hafnium and its compounds. Hence, it is justified to have a heterogeneous collection of outcome measures in the included studies. The extracted data characteristics from the included studies were tabulated (Table 3).

**Table 3:** Characteristics and data of the included studies of this review

Author, year	Country of study	Intervention	Outcomes	Methods of analysis	Relevant findings
Chao Y et al., 2018 [7]	China	Polyethylene glycol (PEG)-modified nanoscale coordination polymers (NCPs) composed of hafnium (Hf4+) and tetrakis(4-carboxyphenyl) porphyrin (TCPP)	Killing cancer cells, likely owing to the interaction of Hf with γ rays emitted from 99mTc to produce charged particles for radiosensitization	Single-photon emission computed tomography (SPECT) imaging	TCPP-PEG NCPs offer exceptional therapeutic results in eliminating tumors with moderate doses of 99mTc after either local or systemic administration. Importantly, those biodegradable NCPs could be rapidly excreted without much long-term body retention.
Kuang Y et al., 2020 [8]	China	Cisplatin-loaded Gd2Hf2O7 nanoparticles (NPs)	1) Combined chemo/ photothermal therapy (PTT)/radiotherapy (RT) in vivo; 2) long-term biodistribution; 3) histology analysis in vivo	Intravenous injection into mice	The less release of Gd showed excellent cytocompatibility, high relaxivity, pH, and dual-sensitive release of loaded cisplatin. The effective PTT/RT ability showed the potential of Gd2Hf2O7@PDA@PEG-Pt-RGD NPs as multimodal theranostic nanoplatform for MRI-guided combined chemo/PTT/RT.
Rajaraman V et al., 2021 [9]	India	Hafnium (Hf)-coated titanium (Ti) and uncoated Ti	Implant stability histologic evaluation of bone formation toxicology	Hematoxylin and eosin (H&E) stain, Masson trichome stain, aspartate aminotransferase (AST), alanine transaminase (ALT), creatinine kinase (CK) assay using enzyme-linked immunosorbent assay (ELISA) kit	Hf coating in the rat mandible showed promising osseointegration with good tissue biocompatibility.
Seweryn A et al., 2020 [11]	Poland	Homogeneous, amorphous layer of Hf(IV) oxide (HfO_2_) using atomic layer deposition (ALD) technology	Pre-osteoblast (MC3T3), pre-osteoclasts (4B12), and macrophages cell lines	Immunofluorescence and reverse transcription-quantitative real-time polymerase chain reaction (RT-qPCR)	HfO_2_ 1) enhanced osteogenesis, 2) reduced osteoclastogenesis, 3) did not elicit an immune response, and 4) exerted anti-inflammatory effects. HfO_2_ layer can be applied to cover the surface of metallic biomaterials in order to enhance the healing process of osteoporotic bone fracture.
Rajaraman V et al., 2020 [12]	India	Chitosan NP and Hf metal-based composite	Cytotoxic effect and antimicrobial activity	Brine shrimp lethality assay and the disc diffusion method	This study substantiates the antimicrobial activity and highlights the possible cytotoxicity of the chitosan and Hf composite.
Sherstiuk AA et al., 2021 [23]	Russia	HfO_2_ NPs coated with oleic acid and a monomethoxypoly(ethylene gglycol)-poly(-caprolactonelycol)-poly(ε-caprolactone) copolymer shell (nanoplatform)	Targeted delivery of chemotherapeutic compounds, imaging, and an enhanced radiotherapy	Cytotoxicity IC_50_ value	X-ray irradiation of cancer cells loaded with a nanoplatform shows a higher death rate than that for cells without NPs.
Li R et al., 2022 [24]	China	Tannic acid (TA) capped Hf disulfide (HfS_2_@TA) nanosheets	Prophylactic and therapeutic effect and potential of oral administration of HfS_2_@TA on dextran sulfate sodium (DSS)-induced acute colitis	NCM460 cells Balb/c mice intravenous/oral administration followed by H&E stain and blood work up	HfS_2_@TA had excellent therapeutic effects, like repair of the intestinal mucosal barrier, restoration of colonic length, and reduction of proinflammatory factor levels.
Fohlerova Z et al., 2019 [25]	Czech Republic	Flat film and nanostructured anodic Hf-oxide films	Cell culture and cell proliferation	Human osteoblast-like MG-63 cells (European Collection Of Authenticated Cell Cultures (ECACC), Salisbury, UK) were used for in vitro characterization of hafnium oxide (HO) films	Nanostructured Hf film absorbed nine times larger amounts of fibronectin and albumin, relatively better initial attachment and significantly promotes the viability of the cells.
Miyazaki T et al., 2018 [27]	Japan	Pure Hf and Ti-xHf alloy x = 20,40,60,80	Zeta potential indicating negative charge on surface, which indicated apatite-forming abilities in simulated body fluid (SBF)	Electrophoretic light scattering zeta potential analyzer (ELSZ, Otsuka Electronics Co., Osaka, Japan) in a connected box-like quartz cell	1) Pure Hf metal enabled formation of apatite on its surfaces and exhibits bone-bonding potential. 2) The apatite-forming ability of Ti-Hf alloys was low at Ti-60Hf.
Bao J et al., 2020 [28]	China	Folic acid (FA) modified nanoscale metal-organic framework (NMOF) of Hf cluster and Mn(III)-porphyrin	In vivo PTT/RT efficiency	H&E staining was performed on the heart, liver, spleen, lung, kidney, and tumor from one mouse in each group	fHMNM held great clinical application potential for targeting the enhancement of MRI/CT/photoacoustic tomography (PAT) imaging modalities and PTT/RT synergistic treatments of cancer.
Bonvalot S et al., 2019 [29]	Multicenter study	HfO_2_ functionalized NP NBTXR3	Proportion of patients with a pathological complete response	Assessed by a central pathology review board following European Organisation for Research and Treatment of Cancer guidelines in the intention-to-treat population full analysis set	NBTXR3 activated by radiotherapy could represent a new treatment option in patients with locally advanced soft-tissue sarcoma of the extremities or trunk wall
Li J et al., 2022 [33]	China	Metal-phenolic nanosensitizer (Hf-PSP-DTC@PLX) integrated via an acid-sensitive hydrogen sulfide (H_2_S) donor (polyethylene glycol-co-polydithiocarbamates, PEG-DTC)	Radiotherapy intensification and immunogenicity	H_2_S-reprogrammed oxygen metabolism	Hf-sensitization could fully utilize the well-preserved oxygen to intensify RT efficacy and activate immunogenicity. Such a synergistic strategy for improvement of oxygenation and oxygen utilization would have great potential in optimizing oxygen-dependent therapeutics.

Risk of Bias

**Figure 2:**
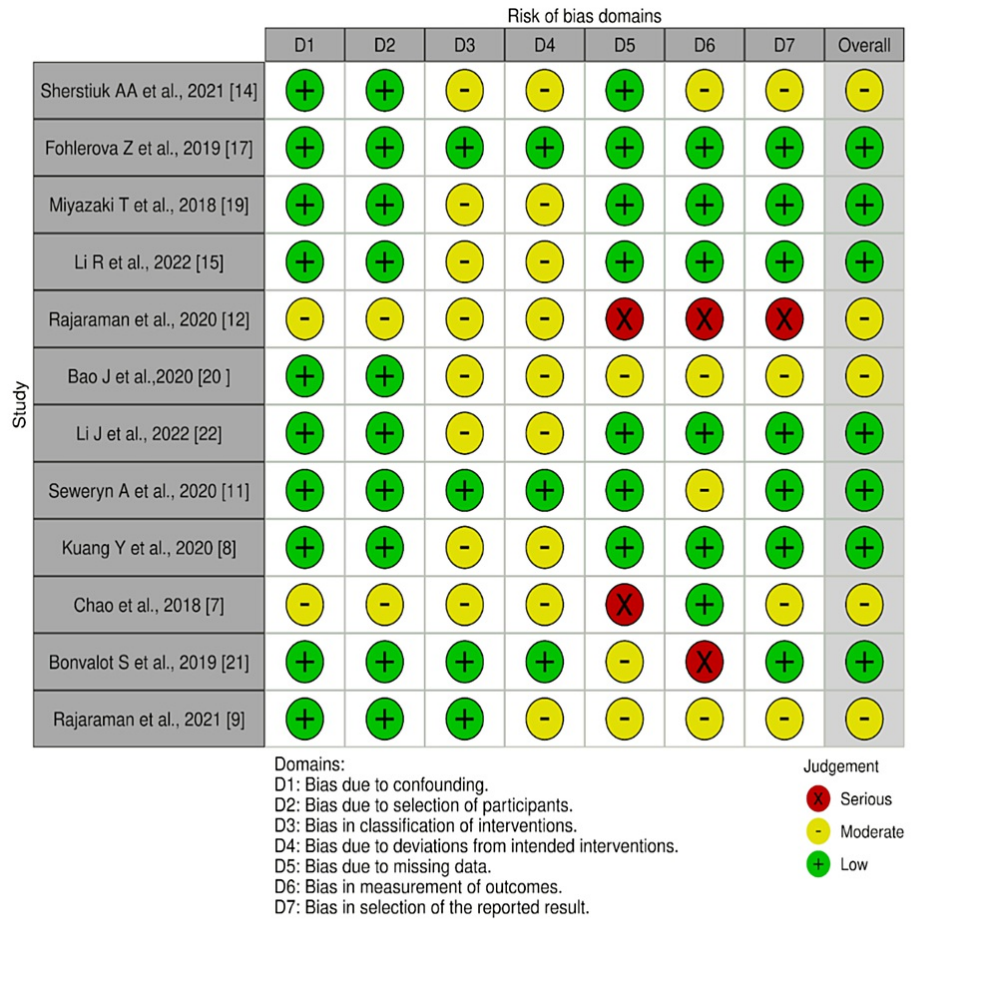
Risk of bias table for the included studies in the systematic review using the ROBINS-I tool by Cochrane Database of Systematic Reviews Red (X) = serious risk of bias. Yellow (-) = moderate risk of bias. Green (+) = low risk of bias.

The risk of bias was assessed using the Risk Of Bias for Non-Randomized Studies: Intervention (ROBINS-I tool) provided in the Cochrane Database of Systematic Reviews (Figure 2).

Discussion

Titanium is used worldwide as a medical biomaterial in prosthetics. Titanium in dentistry is well-established and used in prosthodontics. From metal frameworks to dental implants, titanium plays a vital role in prosthodontics. Finding alternative biomaterials comparable to this metal would be a challenging task. This said, very few scientists have explored different elements in the periodic table to replace this metal [10]. Hafnium is a promising element as it belongs to the same group in the periodic table as that of gold standard titanium [31]. Since the properties of the same group elements are comparable, newer elements as an alternative to titanium could be explored [31-33].

The current study aims to aggregate and critically analyze the studies that discuss biomedical applications of hafnium metal. Our study has collected scientific evidence from articles that highlight the uses of hafnium in various medical disciplines. The widespread use of this transition metal is researched in targeted RT, chemoradiotherapy, inflammatory bowel disease treatment, and bone tissue regeneration [34,35]. In previous studies on RT, hafnium has proven as a potential biomaterial [23,26, 36].

Functionalized hafnium oxide nanoparticles (NBTXR3) have been synthesized to increase the effects of RT [35]. Hafnium-based nanoparticles are potent contrast enhancement agents for imaging in cancer. They are also used for liquid biopsy in diagnosing cancer [36]. In the past two decades, these nanomaterials have grown to be potential biomaterials for two main fields. One is the CT-guided bioimaging and RT-associated cancer treatment due to their excellent electronic structures and intrinsic physiochemical properties [37].

Hafnium has shown promising tissue response and hence cemented its biocompatibility in the research arena [38-40]. Studies also show osseointegration properties exhibited by hafnium coatings over titanium surfaces [10,27]. Research has been done on chemoradiotherapy and the immune therapeutic properties of hafnium. Our previous research on the lines of bone tissue adhesion over hafnium metal or coated hafnium surfaces also showed moderate success [5,9]. This study adds to the existing evidence and analyzes the overall biomedical applications.

On the whole, the biomedical application of the metal hafnium has been often explored in the past decade. The applications are majorly limited to the therapeutic section, especially on cancer. Minor exploration in the field of dentistry suggested that hafnium is biocompatible with positive results in bone tissue integration to dental implants. This review thus provides an overview of the avenues in which the metal hafnium can be explored and experimented with.

## Conclusions

This review outlines an overall bigger picture of the biomedical uses of hafnium metal, a transition element as a potent biomaterial. Various studies conducted in this regard are either primitive or include wider dimensions. Specific research on this metal or its potential applications is in the groundwork. In conclusion, this transition element, hafnium, has some promising scope in the fields of biomedicine with special focus in terms of cancer RT, chemotherapy, and osteogenesis.
